# I’m still me, I’m still a person: war metaphor use and meaning making in women with metastatic breast cancer

**DOI:** 10.1007/s00520-024-08309-5

**Published:** 2024-01-17

**Authors:** Sarah B. Hulse, Zainab Balogun, Margaret Q. Rosenzweig, Anna L. Marsland, Vanessa M. Palmer

**Affiliations:** 1 Department of Psychology, Kenneth P. Dietrich School of Arts and Sciences, University of Pittsburgh, Pittsburgh, PA USA; 2 Division of Breast Surgery, Department of Surgery, University of Pennsylvania, Philadelphia, PA 19104 USA; 3 School of Medicine, University of Pittsburgh, Pittsburgh, PA USA; 4 School of Nursing, University of Pittsburgh, Pittsburgh, PA USA

**Keywords:** Metastatic breast cancer, War metaphor, Meaning making, Narrative medicine

## Abstract

**Purpose:**

The war metaphor is one strategy used frequently in breast cancer to inspire individuals in a “fight” against cancer and assist patients in navigating their illness experience. Despite prominent use, the emotional impact of this language has not been examined in the context of meaning making among women with metastatic breast cancer (MBC).

**Methods:**

This study involved a semi-structured interview considering the war metaphor’s impact on women’s illness experience with MBC. Participants (*n* = 22) had been diagnosed with MBC for at least 6 months or following 1 disease progression and were undergoing treatment at an NCI-designated cancer center in Western Pennsylvania at the time of interview. Each participant underwent an individual interview exploring the war metaphor’s impact on illness experience. Qualitative thematic analysis was performed to assess feelings about the war metaphor and emotional response to the lived experience of cancer.

**Results:**

Two themes were identified surrounding metaphor use and participants’ experiences with meaning making in cancer. First, women with MBC perceive the diagnosis as an “unfair fight” due to its incurable nature. Second, patients use alternative language of “living life” and communicate resistance to being defined by their cancer diagnosis.

**Conclusion:**

War metaphors are one collection of terminology people use to understand their diagnosis. However, their use may apply pressure to prioritize positivity in the face of diagnosis and treatment, in a unique clinical context where this may not be adaptive. These findings affirm a need to consider patients’ lived experiences to best facilitate psychological adjustment to illness.

## Introduction

Emotional distress has been referred to as the “sixth vital sign” of cancer care [1] with negative emotional experiences having implications for cancer treatment and experience of illness, including predicting reduced adherence with medical treatment and oral chemotherapy, decreased quality of life, and elevated risk of mortality [2–7]. Few studies on the psychosocial experience of cancer examine trajectories of emotional response among women with metastatic breast cancer (MBC), which is a life-limiting disease treated with relatively aggressive therapies for many years. Current treatments emphasize the extension of life, making MBC a chronic illness [8]. Patients living with MBC have a wide range of emotional responses to diagnosis, treatment, and other clinically and emotionally significant events in the narrative of disease. This study is interested in how use of the war metaphor (e.g., “You are a fighter”) impacts women with MBC and how they engage with their illness experience emotionally.

Popularized by the 1970s war on cancer campaign, the war metaphor positioned cancer as an enemy to be defeated [9] and has persisted today in framing research, cancer prevention, and treatments as weapons in battle [10]. The war metaphor is widely used by patients, family members, clinicians, and society in discussing responses to cancer. This category of metaphor (e.g., “I am fighting cancer”) offers patients an active role in their experience of illness, which may otherwise feel left to outside determination. Despite prominent use, the war metaphor has faced criticisms for its potential for harm against patients [11–13] and in healthy participants has been found to increase fatalistic beliefs about cancer, make cancer treatment seem more difficult, and make a patient with cancer feel guilty if they do not recover [9, 13]. The literature on MBC patient response to the war metaphor is limited, but criticisms of this metaphor framework are particularly relevant. A war against cancer implies not only two sides in conflict but also the inevitability of a battle’s winner and loser. If the war metaphor pushes women to fight, it then also shames women who do not pursue the same direction with the same hopefulness in response to cancer. Given the terminal implications of MBC, it is necessary to understand how patients with MBC experience the prevalent language of the war metaphor.

This study holds particular interest in the narrative meaning making of life events, a theoretical model proposed to improve understanding of psychological adjustment to life-altering events [14], such as the diagnosis of a chronic condition like MBC. The model relies on the concept of contingency, wherein an individual experiences a crisis of meaning in response to the instigating event and utilizes meaning making to develop a new interpretation of the life event in the greater context of their life narrative. With meaningful reinterpretation, the experience of contingency is reduced, and quality of life is restored. This narrative meaning-making progress is sustained as long as the crisis of meaning continues. Hartog et al. (2020) [14] argue that based on previous literature indicating autobiographical reasoning does not always increase well-being and that denial may be beneficial in the short term, it is possible that narrative meaning making is a tool most impactful in promoting psychological adjustment in response to ongoing life events, such as a chronic illness. This model emphasizes the use of person-driven narratives in successfully approaching the meaning-making process to resolve a crisis of meaning. As narratives are critical to meaning-making processes, metaphors too are critical to narrative construction. In understanding the experiences of patients with metastatic breast cancer, this project seeks to examine emotional impact and patient use of the war metaphor in regulating their emotional response to diagnosis and ongoing treatment. This is an important gap in the literature that must be filled to improve understanding of patient experience and to further guide emotionally supportive care.

## Methods

The data for this analysis was one aspect of a larger clinical study qualitatively comparing and contrasting life experience (i.e., social context, social relationships, physical context, and individual demographics and risk behaviors) of Black and white women with advanced breast cancer. This current project focused on the war metaphor’s impact on emotional experience and thus as an important piece of patients’ broader experience with illness.

### Eligibility and enrollment

Participants were identified via medical chart review based upon diagnosis of MBC and an upcoming clinical appointment at UPMC Breast Cancer Clinic. Eligibility criteria included that participants must be 18 years or older, self-identify as female and either Black or white on their medical record demographic form, and diagnosed with metastatic (stage 4) breast cancer for at least 6 months or have experienced at least one disease progression. We conducted purposeful sampling to ensure variation in age and income to the extent possible in our sample size. Requests made to the clinic team for financial assistance were used as a proxy for low vs. high income. Patients who met the criteria received a letter describing the study and were given the opportunity to provide their phone number. A research associate then contacted interested participants to discuss the study and collect consent via phone.

### Interviews

A doctorally prepared nurse with community education expertise conducted the audiotaped interview with the participant once consented. Each subject’s participation was limited to one interview, lasting approximately one hour, unless additional interviews were required with emerging analytic interpretations. When needed, follow-up interviews were also audio-recorded and transcribed. The semi-structured interview guide was developed and iteratively updated around two areas of interest: (1) social and behavioral needs guided by the National Institute of Minority Health and Disparities model and (2) the war metaphor and language surrounding breast cancer. Participants were compensated for their time.

### Qualitative analysis

Investigators transcribed the audiotaped interviews verbatim and utilized a cross-case, framework thematic analysis using the Iterative Categorization technique [15]. Following each session, the interviewer and two members of the research team wrote memos to describe any salient aspects of the participant’s responses, demeanor, context, or setting, and record personal reflections on the process or content of the interview encounter. Thematic analysis then focused on the transcribed audiotaped interviews. For purposes of analysis, participants were identified by their assigned research identification number. The use of the war metaphor throughout the course of the interview, prior to direct questioning about the war metaphor, was analyzed alongside detailed analysis of participants’ descriptions of their illness experience. Narrative analysis was used to categorize how patients described the metaphor through individual responses. Constant comparative analysis between two readers was performed to answer the research question.

## Results

### Sample

The study sample is characterized in Table 1. A total of 22 women completed the study interviews, with an additional 5 women who consented to participate but did not complete the interviews. Participants’ median age was 54.63 (range: 26–72). Clinically, participants were classified as stage 4 (metastatic) breast cancer and had been diagnosed for a mean of 49.14 months (range: 7–107) at the time of interview. All participants were either on second-line treatment or beyond or had been diagnosed with metastatic breast cancer for at least 6 months, and participants were predominantly (40.9%) accepting chemotherapy treatment at the time of interview.

**Table 1:** Descriptive statistics for demographic characteristics

	*n* (%)	Mean ± SD	Min	Max
Age (years)		54.63 ± [10.21]	26	72
Race				
Black or African American	6 (27.3)			
White	16 (72.7)			
Marital status				
Married	8 (36.36)			
Single	13 (59.09)			
Unknown	1 (4.55)			
HER2 status^1^				
HER2 negative	12 (54.55)			
HER2 positive	9 (40.91)			
Unknown	1 (4.54)			
Insurance				
No insurance or out of state	6 (27.27)			
Public	7 (31.82)			
Private	7 (31.82)			
Unknown	2 (9.09)			
Requests for financial assistance				
Requested	6 (27.27)			
Not requested	16 (72.73)			
Treatment at interview				
Chemotherapy	9 (40.91)			
Endocrine	2 (9.09)			
CD4/6 inhibition	6 (27.27)			
Herceptin therapy	4 (18.18)			
Unknown	1 (4.55)			

^1^All participants with complete medical records (*N* = 21) were estrogen positive

### The war metaphor in the lived experience of metastatic breast cancer

In understanding the complex interactions between the war metaphor and emotion in the lived experience of metastatic breast cancer, this study considers meaning-making processes associated with war metaphor use as well as preferred alternate terminology that arose in the setting of qualitative interviews. The thematic analysis of qualitative data identified that two core themes, “unfair fight” and “living life,” were instrumental to meaning making in women’s descriptions of their lived experience with metastatic breast cancer.

#### Unfair fight

One prevailing theme indicates that the fight implicated in the war metaphor of metastatic disease differs for these women from the *fight against cancer* in a meaningful way. Participants frequently noted the incurable nature of metastatic breast cancer, not the cancer itself, as the factor causing greatest emotional distress, with one participant stating, “They say it’s not curable. It’s treatable but not curable...I mean, it could be going through all of this and nothing may work at all, so the most distressing thing is thinking that. Um, how much time do I have?” Another participant echoed this sentiment: “[The most distressing thing is] that it will never go away.” This participant had previously stated that her diagnosis with MBC had not changed her life, and that she has been able to continue living her daily life as before diagnosis, yet the chronic nature of metastatic disease still caused distress. Relatedly, the uncertain prognosis of illness was salient to several participants, who expressed distress about the inability to make future plans. “I’m hesitant to make plans, too far out because you know the idea before of setting up a vacation a year in advance was nothing. Now, it’s like I’m not gonna be well enough to do that.”

The chronic nature of metastatic breast cancer served as an isolating factor from breast cancer communities for several women. One participant expressed, “I found almost like the isolation comes from the survivor community of breast cancer...like women who have, you know, were maybe in early-stage diagnosis and they went through the chemo and now they’re, you know, quotations cured. Um and they don’t like to hear about the metastatic aspect of it.” Several participants shared this sentiment, particularly noting the month of October, breast cancer awareness, and pink branding as isolating and not resonating with their lived experiences of illness.

Participants’ unique awareness of their life span as being relevant to their experience with metastatic breast cancer was a recurring theme. One woman stated, “When you have cancer, you’re always going to have in the back of your mind thinking you know how long do I have? But you don’t really have a choice in that, so you just move on.” Several participants repeated this terminology of being constantly aware of a terminal prognosis “in the back of your mind.” Another participant shared, “You know breast cancer, particularly metastatic breast cancer, because you know you will be in treatment for the rest of your life, it can feel isolating ‘cause you can’t – you can’t control it, and you’re not going to be done with it until you, until you’re really done.” Several participants described this awareness as knowing their “expiration date.” One participant using this term expressed the severity of this on her daily life: “...and that’s why I actually started to see a therapist because that was kind of in my mind constantly.”

Perceptions of metastatic breast cancer as an unfair battle are integrated with these feelings and concerns, as one woman described, “...to me it’s a battle that you have to fight but I don’t I – it’s to me, it’s an unfair battle. I mean you know you’re dependent on medications and so many things I think you have to fight it.” For several participants, the view of metastatic disease as an unfair fight originated from obituaries of friends and loved ones who had died from metastatic breast cancer. One participant said, “...I just don’t like the war mentality of it ’cause it’s an unfair battle, I guess...I also don’t like when people say, ‘she was stolen by’ or ‘taken by’...or ‘she lost.’ I think that’s the one that really bothers me, ‘she lost the battle.’” One participant described, “...the problem is that you know when you talk of ‘battles’ and ‘wars’ and ‘fighters’ and ‘survivors,’ that means that there’s a loser somewhere in it and...I don’t like to think of people as having lost it because they did the best they could.” Another women stated, “I know when I eventually die, I don’t want people saying I lost.” Instead, several participants expressed wishing to be remembered for their personal characteristics, including one woman who stated she wanted to be remembered as “keeping hope and trying to live as normal as possible.”

#### Living life

New terminology emerged in our data that participants used both alongside the language of the war metaphor as well as when countering the language of the war metaphor. Participants emphasized the experience of living with metastatic breast cancer rather than fighting it. One woman shared, “I can’t fight having cancer, it’s already happened so, all I can do is try to live well with it.” Another participant stated, “There’s no warrior, there’s no kicking its butt, there’s no, you know what I mean? It’s just living and pray that the treatment doesn’t stop working.” One woman suggested that her feelings about the war language were related to the metastatic nature of her diagnosis: “I think if I had a lower grade cancer and you know I had that endpoint in my mind, I might [use war metaphors], but because it’s metastatic and I know it’s just kind of, you know I’m not – I’m not going to beat it...I’m just gonna live with it every day.”

Several women also emphasized the importance of living with normalcy in their daily lives. One participant described, “...it took time, but just the acceptance allows me to be able to try to somewhat live a normal life.” In offering advice to other women with metastatic breast cancer, another participant echoed this sentiment while emphasizing the importance of emotion expression and acceptance: “Accept the diagnosis and be able to talk about it. And just try to continue to live a normal life.” Another woman stated her preference for alternative language in learning to live with metastatic illness: “I feel that it’s a challenge; it’s not so much a battle. It’s a pretty serious challenge, but it kind of- it becomes part of your life I guess.”

Related to the experience of living life with normalcy, several participants referenced a desire to be acknowledged as a whole person outside of their diagnosis. One participant described her experience returning to work after being on temporary leave for several months after her diagnosis. Her colleagues prepared a fundraiser for breast cancer awareness and decorated the workspace in pink signage and linens. She expressed: “I was struggling with it and here it was everywhere I walked. Which is this blaring cancer cancer cancer...I know it came from a place of love and support but it also made me feel reduced to...just one thing.” Another woman concluded: “I don’t want to be the disease. I’m still me. I’m still a person.”

## Discussion

This study presents an initial thematic analysis of lived experiences in metastatic breast cancer and its use in meaning-making processes. The inquiry investigated the role of war metaphor terminology in patients’ lived experiences, both in how this language is shaped by social expectations of patient’s lived experience of breast cancer and how it contributed to meaning making in response to emotional distress. The view of metastatic illness as an unfair battle was prominent among participants, who acknowledged their dependence on medication and their lack of choice in diagnosis. Several women expressed a particular dislike for the phrase “she lost the battle,” which is used colloquially to describe the deaths of women with breast cancer. Prior work [16] suggests the war metaphor can be used by patients to feel a sense of control over cancer outcomes and may thus be unhelpful to patients who feel a lack of control, such as patients experiencing metastatic disease or progression deemed incurable. The war metaphor then attributes personal responsibility to those who bear this language, communicating to patients that disease progression is a personal failure or loss. Of note, some participants acknowledged feeling the positivity enforced through the war metaphor was not authentic to their illness experience. Women expressed several reasons they did not identify with the war metaphor, including feeling as if everyone was fighting in life, patients were doing what needs to be done, or patients were not the ones in control of their illness (i.e., medical professionals, medications, or a higher power were responsible for the illness’ trajectory). Concerns about the incurable nature and uncertain prognosis contributed to these feelings. Although it has been suggested that the war metaphor uniquely allows women with metastatic breast cancer to communicate their lived experiences via revealing the difficulty of having cancer and “collapsing in battle” [17], use of the war metaphor may prove inauthentic to some women’s experience. For women who do not identify with the war metaphor, it may be more distressing to interpret their illness experience in a manner that aligns with such language. Notably, this also allows for use of the war metaphor to be useful to meaning making if it is authentic to the experiences of the patients using it, as some women with breast cancer may feel. Our results affirm the importance of encouraging authentic emotion expression in patients with metastatic breast cancer, who may experience pressures to portray strength and positivity.

The identified themes suggest the experience of metastatic illness is emotionally different from that of early-stage breast cancer due to the diagnosis’ chronic and ultimately fatal implications. These differences were also evident in the 2021 work of Ciria-Suarez and colleagues [18] in the thematic analysis of the breast cancer lived experience, from initial diagnosis through metastatic breast cancer, including the feelings of isolation from women with early-stage disease, intensified by the commercialized pink washing evident in the month of October. Patients regularly referenced the incurable nature of metastatic breast cancer as causing them emotional distress even as they attempted to learn to live alongside this diagnosis. The uncertain prognosis of metastatic breast cancer was a recurrent theme in defining participants’ emotional response to disease. Participants communicated worries surrounding their ability to complete future tasks or have desired time with their families. The uncertainty of metastatic breast cancer prognosis creates an emotion-eliciting situation in which features of the illness experience (i.e., time to enjoy with family or ability to plan for the future) are left unclear [19]. This suggests a potential disruption to meaning making, as patients struggle to interpret features of their diagnosis and resolve its threat to their emotional well-being.

New terminology of “living life” emerged in this analysis. Implicit in this language of “living life” is seeking and finding meaning in their personal lived experience with metastatic breast cancer. Meaning making is an adaptive strategy with regard to the chronic nature of this illness and differs in its value from those with an early-stage diagnosis, wherein autobiographical reasoning may be an unnecessarily taxing process [14]. Contrary to the war-like language of “unfair battle,” “living life” suggests successful integration of chronic illness into one’s life experience and thus an emotional resolution to contingency (i.e., diagnosis of metastatic breast cancer). Faced with long-term treatments and a stable or increasing symptom profile, patients with metastatic disease employ this narrative to accept the aspects of their lived experience that are beyond their perceived control. Although women were resistant to being defined by their cancer, this language also suggests a narrative of living not in spite of but alongside their illness. “Living life” and living with normalcy were expressed goals of several participants in learning to accept their diagnosis and incorporate it into their greater life narrative.

### Study limitations

There are a few limitations attributed to the nature of this study. First, the study population was limited to the demographics of Magee-Womens Cancer Center, and our participants were predominantly high income and insured. Although the small sample size and interview methodology allow for thematic analysis and an understanding of patient narratives, it is not representative of all patients with metastatic breast cancer or breast cancer in general.

Of note, our study only included qualitative interviews with women with metastatic breast cancer, so any comparisons of frequency of this language in women with early-stage illness versus with advanced-stage illness are predictive. This study is thus limited in its representation of experiences with cancer and does not represent all patient experiences. Future studies may consider a more direct comparison through sampling from patient populations of various disease staging. This has been partially examined in previous literature by Bodd et al. (2022) [16], which supports the significance of language in the meaning-making process in which patients with cancer integrate these lived illness experiences into their broader life narratives.

## Conclusion

This study takes an analytical view of the war metaphor in describing its rhetorical implications for patients with metastatic disease but recognizes the unique perspective of each patient and their narrative. Until clinicians are able to collaboratively define a common language with patients on the basis of their individual preferences, clinicians should avoid using the war metaphor in conversions in clinical settings to not create unnecessary distress. Metaphors may be a powerful tool in developing this common language and facilitating the relationship between patient and clinician. More research is needed to examine the impact of metaphorical language on the lived experiences of patients with both metastatic and early-stage cancers.

## Abbreviation

MBC: Metastatic breast cancer
